# Effects of sodium-glucose co-transporter 2 (SGLT2) inhibitors on renal outcomes in patients with type 2 diabetes mellitus and chronic kidney disease: A protocol for systematic review and meta-analysis

**DOI:** 10.1097/MD.0000000000024655

**Published:** 2021-02-26

**Authors:** Baisong Yu, ChunXia Dong, ZhiJuan Hu, Bing Liu

**Affiliations:** Department of Nephrology, Hebei General Hospital, Shijiazhuang, Hebei, China.

**Keywords:** chronic kidney disease, meta-analysis, renal outcomes, SGLT2 inhibitors, type 2 diabetes mellitus

## Abstract

**Background:**

Many studies have shown the effects of SGLT2 inhibitors on type 2 diabetes, but the effects in patients with type 2 diabetes with chronic kidney disease remains unclear. This study aims to evaluate the effects of SGLT2 inhibitors on renal outcomes in patients with type 2 diabetes mellitus with chronic kidney disease.

**Methods:**

We conducted systematic searches of PubMed, Embase, and Cochrane Central Register of Controlled Trials up to April 30, 2020 and included randomized controlled trials of SGLT2 inhibitors in adult type 2 diabetes mellitus (T2DM) patients with chronic kidney disease (CKD) reporting estimated glomerular filtration rate (eGFR) and/or urine albumin/creatinine ratio (UACR) changes and/or acute kidney injury or failure (AKI). Random effects models were adopted to measure the pooled outcomes.

**Results:**

Nine studies with 8826 participants were included. SGLT2 inhibitors were not associated with a significant change in eGFR (mean difference (MD), −0.75 ml/minutes per 1.73 m^2^, 95% CI −1.61 to 0.10, *P* = .09) in type 2 diabetic patients with CKD. UACR reduction after SGLT2 inhibitors was significant in type 2 diabetic patients with CKD (MD −24.27 mg/g, 95% CI −44.46 to −4.09, *P* = .02). SGLT2 inhibitors associated with AKI in the patients were significant (OR 0.80, 95% CI [0.66 to 0.98], *P* = .03).

**Conclusion:**

SGLT2 inhibitors had no significant effect on kidney function (eGFR measured) in the pooled analysis. And SGLT2 inhibitors effectively reduced UACR in T2DM with CKD. Besides, SGLT2 inhibitors could reduce the incidence of AKI.

## 1 Introduction

Diabetes mellitus (DM) have affected more than 415 million adults worldwide. It is estimated that more than 640 million adults will be affected by DM by 2040.^[1]^ About 35% of type 2 diabetes mellitus (T2DM) develop into kidney disease.^[2]^ Diabetic nephropathy has largely increased the incidence and prevalence of the end stage renal disease (ESRD).^[3,4]^ However, current strategies in treating diabetic nephropathy including glycemic control, blood pressure control, avoidance of nephrotoxic agents and so on.^[5]^ Therefore, it is essential to develop effective therapies to prevent progression of diabetic nephropathy.

Sodium-glucose co-transporter 2 (SGLT2) inhibitors are a new class of antihyperglycemic drugs, which lower blood glucose by blocking glucose reabsorption via inhibiting SGLT2 at the proximal renal tubule. SGLT2 inhibitors are becoming more and more popular because of their multiple benefits. In addition to glycemic control, SGLT2 inhibitors can also lower body weight and blood pressure and reduce relevant adverse cardiovascular consequnces in T2DM patients with high cardiovascular risk.^[6–9]^

SGLT2 inhibitors can protect the kidney. The complicated mechanisms have not been illustrated clearly yet. One of the most important mechanisms is that SGLT2 inhibitors block glucose and sodium reabsorption in proximal tubule; and increase sodium transport to the macula densa, thus activating tubuloglomerular feedback (TGF) and causing afferent arteriolar vasoconstriction. In this way, SGLT2 inhibitors can relieve long-term glomerular pressure, reduce albuminuria and slow down the decline of renal function.^[10–13]^ Luseoglifozin is a kind of SGLT2 inhibitor, which inhibits the expression of hypoxia-inducible factor (HIF-1) target gene and plays an important role in hypoxia-induced tubulointerstitial fibrosis.^[14]^ The increased of Pin1 in diabeitic mice can lead to infammation and fibrosis of several tissues, such as glomerular mesangial cells and podocyte, while podocytes are normalized by canaglifozin. In addition, canaglifozin can also induce the activation of AMP-activated kinase (AMPK) activation, which inhibits proliferation of mesangial cell and plays an anti-infammatory role in hematopoietic cells.^[15]^ More mechanisms need to be explored in the future.

Many clinical trials have reported kidney-related outcomes after the use of SGLT2 inhibitors.^[8,9,16,17]^ At present, there is no systematic review on whether SGLT2 inhibitors can preserve renal function, reduce UACR, and decrease adverse effects in patients with T2DM and CKD. Therefore, we conducted a meta-analysis of this randomized controlled trials (RCTs) to determine the effects of SGLT2 inhibitors on eGFR, UACR and AKI compared to with placebo or other antidiabetic treatments in patients with T2DM and CKD.

## 2 Materials and methods

This study is a systematic review and meta-analysis to assess the role of SGLT2 inhibitors in patients with T2DM and CKD compared to placebo or positive controls. It is conducted and reported in accordance with the PRISMA (Preferred Reporting Items for Systematic Reviews and Meta-Analyses) Guidelines.^[18]^

### 2.1 Study selection

Two reviewers (B.Y. and C.D.) independently screened the search results and retrieved relevant studies for further evaluation. The retrieved full-text articles were examined in parallel by 2 reviewers (B.Y. and C.D.) for inclusion according to predetermined criteria. We included RCTs conducted on adult patients with T2DM and CKD (defined as eGFR < 90 ml/minutes/1.73 m^2^) comparing SGLT2 inhibitors with placebo or other antidiabetic drugs, and reporting changes in eGFR and/or UACR and/or AKI. Only manuscripts published in English were included. For multiple papers in the same study, only the first report on renal outcomes was included. Abstracts, case reports, letters, reviews and those not reporting outcomes of interest were excluded. Disagreement was resolved through discussion and/or consultation with the third reviewer (Z.H.).

### 2.2 Data extraction and validity assessment

The 2 reviewers (YBS and DCX) independently used standard data extraction tools to record the following properties of each study: study characteristics (author, year, study design, randomized method and follow-up duration), participant characteristics (sample size, age, course of diabetes, baseline HbA1C levels, baseline blood pressure, eGFR and UACR), therapeutic intervention (type, dose and duration of SGLT2 inhibitor), control group (placebo-controlled or active-controlled), outcomes of interest (means and standard deviations (SDs) of changes in eGFR, UACR in treatment and control groups)and odds ratio (OR) in acute kidney injury or failure in treatment and control groups).

### 2.3 Risk of bias assessment

The quality of the study was evaluated by 2 authors (YBS and DCX) independently using the “Risk of bias” assessment tool from the *Cochrane Handbook for Systematic Reviews of Interventions*, version 5.1 (2011). The domains of assessment included random sequence generation, allocation concealment, blinding of participants and personnel, blinding of outcome assessment, incomplete outcome data, selective reporting and other bias.

### 2.4 Statistical analysis

For dichotomous data such as AKI, OR with 95% confidence intervals (CI) were adopted for assessment. For continuous data such as eGFR and UACR, the MD and the 95% CI were calculated. For studies in which SD was not directly reported, SD was calculated from standard error (SE) or 95% CI, or Inter Quartile Range (IQR). As clinical and statistical heterogeneity were anticipated, we decided to use a prior random-effect model in data synthesis.

Statistical heterogeneity was quantified using the Cochrane *Q* test and *I*^*2*^ statistic, where *I*^2^ values >50% indicated substantial heterogeneity. When significant heterogeneity was indicated, we tried to determine the potential reasons. Subgroup analysis was conducted based on baseline eGFR levels of patients. Test for subgroup differences were carried out using RevMan 5.3 (The Nordic Cochrane Centre, The Cochrane Collaboration).

Statistical analyses were performed using the RevMan 5.3 (The Nordic Cochrane Centre, The Cochrane Collaboration). Statistical significance was set at *P* < .05 for all analyses.

### 2.5 Statement of ethics

All analyses were based on previous published studies, thus no ethical approval and patient consent are required.

## 3 Results

### 3.1 Study characteristics

**Figure 1:**
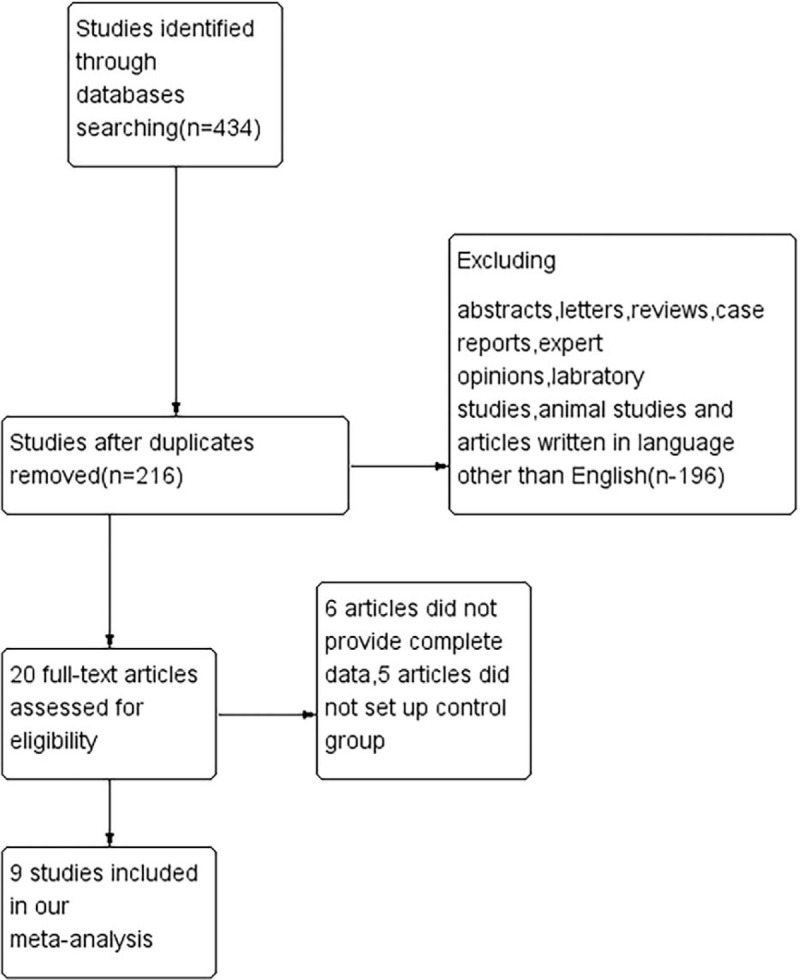
The flow diagram.

After initial literature search, 434 potentially relevant publications were identified in PubMed, Embase, and Cochrane Central Register of Controlled Trials, Of which, 9 studies involving 17 trials, which met our inclusion criteria and were selected for meta-analysis.^[16,17,19–25]^ The flow chart was shown in Figure 1. Efficacy and safety of SGLT2 inhibitors were compared with placebo or other anti-diabetic agents. Of these, 1 trial included 3 different doses of SGLT2 inhibitors,^[23]^ 3 trials contained 2 different doses of SGLT2 inhibitors^[20–22]^ and 4 trials contained only 1 dose of SGLT2 inhibitor.^[16,17,19,25]^ One trial included groups in different stages of CKD.^[24]^ We included these results in pooled analysis and regarded them as different comparisons. Basic characteristics of included trials and demographic data of participants were presented in Table 1. Dapagliflozin, empagliflozin, canagliflozin and bexagliflozin were SGLT2 inhibitors used as treatment drugs. Placebo was commonly used in 17 trials as control group. All trials lasted for over 24 weeks. And 2 trials lasted for over 2 years.^[16,17]^ All trials were multicenter studies. The total number of participants was 8826, ranging from 40 to 4401 across different trials. The mean age of patients in all trials was over 55. All patients were previously diagnosed with T2DM and most of them received background anti-diabetic medications except for intervention therapies.

**Table 1:** The basic characteristics of the enrolled studies.

Study	Dose (mg)	Control group	Durati-on of follow up	Sample size	Mean age (years)	Mean duration Of diabetes (years)	Mean baseline HbA!C (%)	Mean baseline blood pressure (mmHg)	Mean baseline eGFR (ml/min/1.73m2)	Mean baseline urine ACR(mg/g)	Outcom-e
Canagliflozin											
Takashima et al (2018)^[19]^	100 mg	placebo	52 weeks	40	65.1	NR	7.5	139/79	56.3	149	eGFR, UACR
Yale et al (2014)^[20]^	100 mg	placebo	52 weeks	269	68.5	16.3	8	NR	39.4	256.8	eGFR, UACR
	300mg										
Bode et al (2013)^[21]^	100 mg	placebo	104 weeks	714	63.6	11.7	7.7	131.0/75.7	77.5	NR	eGFR
	300 mg										
CREDENCE^[16]^	100 mg	placebo	2.62years	4401	63	15.8	8.3	140.0/78.3	56.2	1074.3	AKI
Dapagliflozin											
Kohan et al (2014) ^[22]^	5 mg	placebo	104 weeks	252	67	16.9	8.35	132.1/73.3	44.6	73	eGFR, UACR, AKI
	10 mg										
Wilding et al (2012)^[23]^	2.5 mg	placebo	48 weeks	800	59.3	13.6	8.53	138.5/80.1	78.4	75.2	eGFR, UACR
	5 mg										
	10 mg										
Empagliflozin											
Barnett et al (2015)^[24]^	10 mg	placebo	52 weeks	290	62.6	NR	8.03	135.3/76.9	71.6	NR	eGFR
	25 mg										
	25 mg	placebo	52 weeks	374	64.9	NR	7.96	133.7/76.7	44.9	NR	eGFR
Wanner et al (2018)^[17]^	10 mg 25 mg	placebo	3.1years	1819	67.1	NR	8.05	136.25/74.55	48.5	NR	AKI
Bexagliflflozin											
Allegretti et al (2019)^[25]^	20 mg	placebo	24 weeks	312	69.6	15.91	7.98	136.8/NR	45.11	NR	AKI

AKI = acute kidney injury or failure, eGFR = estimated glomerular filtration rate, UACR = urine albumin/creatinine ratio.

### 3.2 The risk of bias

**Figure 2:**
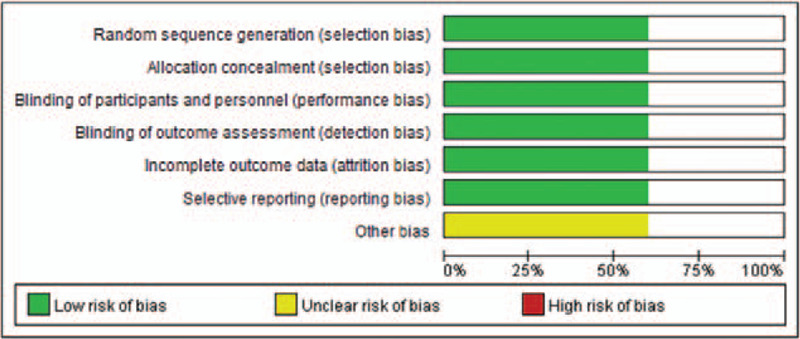
Risk of bias.

Using the criteria recommended in the Cochrane handbook 6, the risk of bias in identified trials was assessed in 9 domains. For all evaluated items, most trials were rated as low risk. All trials were RCTs, in which the process of random sequence generation, allocation concealment, and blinding were described. Therefore, unclear risk was determined in other bias in this study (shown in Fig. 2).

### 3.3 Clinical outcomes

#### 3.3.1 Estimated glomerular filtration rate (eGFR)

**Figure 3:**
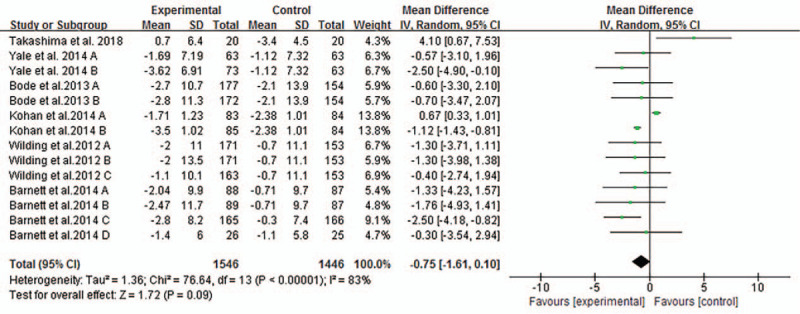
Forest plot for the mean difference of the change in eGFR comparing SGLT2 inhibitors with placebo or other antiglycemic agents. CI = confidence interval, eGFR = estimated glomerular filtration rate, IV = inverse variance, SD = standard deviation, SGLT2 = sodium glucose cotransporter 2.

**Figure 4:**
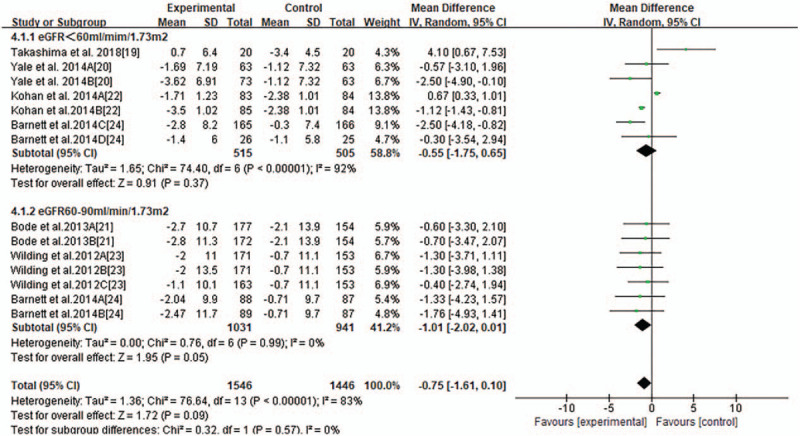
Subgroup analysis of the effect of SGLT2 inhibition on eGFR.

**Figure 5:**
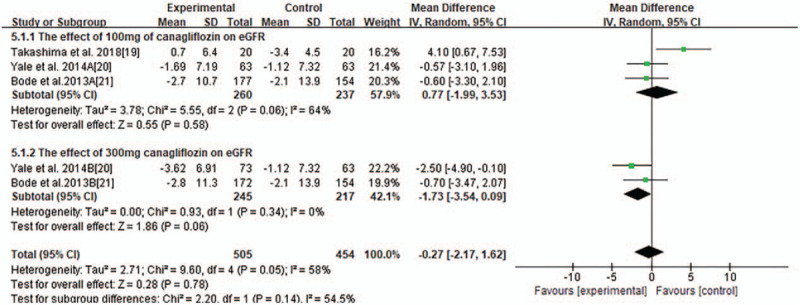
Forest plot for the mean difference of the change in eGFR comparing 100 mg canagliflozin with 300 mg canagliflozin.

**Figure 6:**
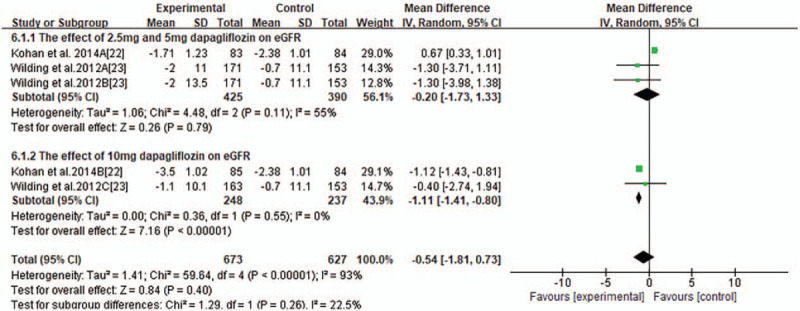
Forest plot for the mean difference of the change in eGFR comparing 2.5 mg, 5 mg dapagliflozin with10 mg dapagliflozin.

**Figure 7:**
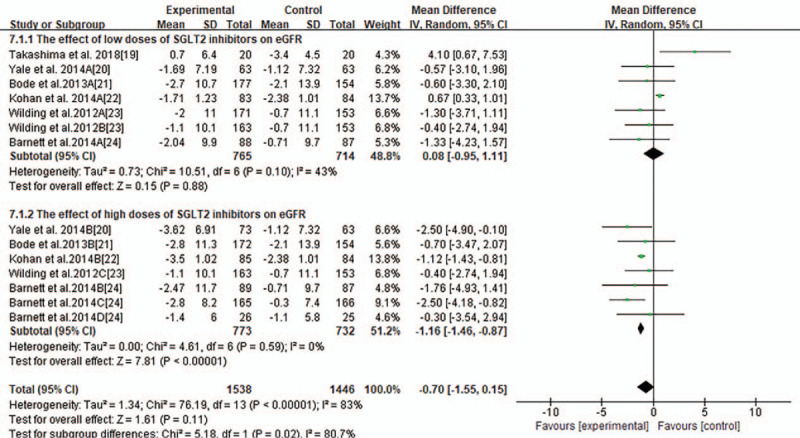
Forest plot for the mean difference of the change in eGFR comparing low-dose SGLT2 inhibitors with high-dose SGLT2 inhibitors.

Most trials reported changes of eGFR of participants from baseline to endpoint and MD of the change ± SD. Pooled analysis showed that there was no significant changes of eGFR associated with SGLT2 inhibitors compared with placebo or other anti-diabetic medications (MD-0.75 ml/minutes/1.73 m^2^, 95% CI −1.61to 0.10, *P* = .09, shown in Fig. 3) in T2DM with CKD. Heterogeneity was substantial between studies (*I*^2^ = 83%, *P* < .00001). To further investigate the possible influencing factors, we conducted a subgroup analysis based on baseline eGFR of participants and found that there was no significant difference in subgroup eGFR (shown in Fig. 4). Besides, we included the studies, in which canagliflozin had 2 doses of 100 mg and 300 mg, dapagliflozin had 3 doses of 2.5 mg, 5 mg, and 10 mg, and empagliflozin had 10 mg and 25 mg. In order to discover the effect of different doses of SGLT2 inhibitors on eGFR, we conducted subgroup analyses. There were no significant changes of eGFR associated with different doses of canagliflozin (shown in Fig. 5). A subgroup analysis of dapagliflozin found that 5 mg of dapagliflozin had no significant effect on eGFR, but 10 mg can cause eGFR to decrease (shown in Fig. 6). A subgroup analysis of empagliflozin was not performed because there was only 1 study of empagliflozin on eGFR.100 mg canagliflozin, 2.5 mg and 5 mg dapagliflozin, 10 mg empagliflozin were considered as low-dose SGLT2 inhibitors, while 300 mg canagliflozin, 10 mg dapagliflozin, 25 mg empagliflozin were high-dose SGLT2 inhibitors. Therefore, we performed subgroup analysis according to low doses and high doses and found that low doses had no significant effect on eGFR. However, high dose had the opposite effect (shown in Fig. 7).

### 3.4 Urine albumin/creatinine ratio (UACR)

**Figure 8:**
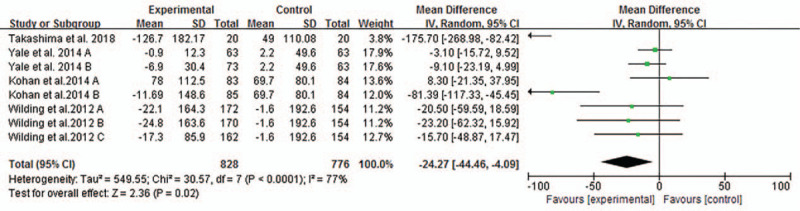
Forest plot for the mean difference of the change in urine albumin-creatinine ratio comparing SGLT2 inhibitors with placebo or other antiglycemic agents.

Four studies reported changes of UACR of participants. Pooled analysis showed that significant changes of UACR in patients with T2DM and CKD after using SGLT2 inhibitors compared with placebo or other anti-diabetic medications (MD −24.27 mg/g, 95% CI −44.46 to −4.09, *P* = .02, shown in Fig. 8)

### 3.5 Acute kidney injury or failure

**Figure 9:**
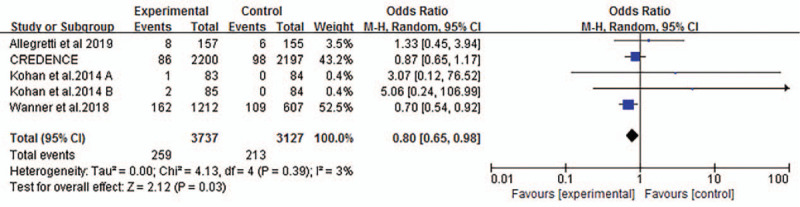
Forest plot for meta-analysis of the association between SGLT2 inhibitors and acute renal injury or failure.

Four studies reported events of acute kidney injury or failure in T2DM and CKD. The incidence of patients who received SGLT2 inhibitors was slightly lower than that of patients in control group (OR 0.80, 95% CI 0.65 to 0.98, *P* = .03, shown in Fig. 9).

### 3.6 Sensitivity analysis and publication bias

Our analyses were robust in both the selection of models and the statistical methods. The substitution of a random effects model for a fixed model did not change our initial qualitative interpretation of the pooled treatment effect of SGLT2 inhibitors on renal function, UACR and AKI. Nine studies were inadequate to detect publication bias.

## 4 Discussion

In this systematic review and meta-analysis, we found that SGLT2 inhibitors had no significant effect on eGFR in type 2 diabetic patients with CKD. Subgroup analysis suggested that there was no significant change in SGLT2 inhibitors with different eGFR levels, though some studies found that dipping of eGFR in shorter trials and preservation of eGFR in trials of longer duration. UACR reduction after SGLT2 inhibitors was statistically significant in type 2 diabetic patients with CKD. Incidence of acute kidney injury or failure was indicated to be lower in patients receiving SGLT2 inhibitors in our study.

In our meta-analysis, we identified SGLT2 inhibitors had no statistically significant effect on eGFR in type 2 diabetic patients with CKD. And heterogeneous source was not eGFR via our subgroup analysis. Although it was found that high-dose SGLT2 inhibitors had an effect on eGFR, fewer studies were included in our article. Hence, more and longer researches of high-dose SGLT2 inhibitors on eGFR were needed. And we noticed a phenomenon was that eGFR reduced in the short-term studies and preserved in the longer-term studies in T2DM with CKD, as has been reported in several clinical trials.^[17,25]^ Like EMPA-REG OUTCOME trial,^[17]^ eGFR decreased for a short time from the baseline to week 4 in the empagliflozin groups. However, during long-term administration, the eGFR remained stable in the empagliflozin groups and declined steadily in the placebo group. Eventually, eGFR increased after cessation the drug in the empagliflozin groups. Similarly, in the CREDENCE^[16]^ study, during the first 3 weeks, there was a greater reduction in eGFR in the canagliflozin group than in the placebo group, subsequently, the decline in eGFR was lower in the canagliflozin group. The phenomenon suggests that initial reduction of eGFR is possibly caused by hemodynamic changes. In our meta-analysis, albuminuria was improved in type 2 diabetic patients with CKD using SGLT2 inhibitors. Our results, in accord with findings of previous meta-analysis,^[26]^ demonstrated the role of SGLT2 inhibitors in slowing the progression of albuminuria. However, we did not conduct the subgroup analysis due to substantial heterogeneity of a few results (*I*^2^ = 77%, *P* < .00001). More recently, results from CREDENCE^[16]^ not only provided solid evidence that canagliflozin reduce the risk of doubling of serum creatinine level, ESKD, eGFR < 15 ml/minutes/1.73 m^2^, dialysis initiated or kidney transplantation and so on, but also showed the decline of UACR in patients with canagliflozin, regardless of their baseline status of albuminuria.

Regarding acute kidney injury or failure of SGLT2 inhibitors in T2DM with CKD, we found that the incidence was slightly lower compared with control group which was also indicated in Cahn trial.^[27]^ In our meta-analysis, there were 2 groups in the EMPA-REG OUTCOME trial^[17]^ and we selected group with eGFR < 59 ml/minutes/1.73 m^2^. Only the outcomes in eGFR < 60 ml/ minutes/1.73 m^2^ were found. Though the studies included were small, this implied that SGLT2 inhibitors could decrease the incidence rate of acute kidney injury or failure to some extent.

Despite rigorous methodology, our study has several limitations. First, we included 9 studies of T2DM with CKD, which might lead to insufficient powerd evaluation. Second, there were only data on 4 kinds of SGLT2 inhibitors (canagliflozin, dapagliflozin, empagliflozin, bexagliflflozin), not all drugs. Third, our study used surrogate endpoints, including eGFR, UACR and AKI, rather than hard endpoints, such as progression of nephropathy or renal mortality.

In conclusion, SGLT2 inhibitors were not associated with significant changes in eGFR in type 2 diabetic patients with CKD in the integrated analysis. And SGLT2 inhibitors were associated with UACR reduction in T2DM with CKD. In the meantime, SGLT2 inhibitors could reduce the incidence rate of acute kidney injury or failure. Further studies are warranted for the renal effects of SGLT2 inhibitors and delaying the progression of T2DM with CKD.

## Author contributions

B.Y. and C.D.: collected and reviewed the articles. B.Y., C.D. and Z.H.: contributed substantially to the analysis and interpretation of data. B.Y.: contributed to manuscript writing. B.Y., C.D., Z.H. and B.L.: involved in drafting and revising the manuscript and gave final approval of the version to be published.

**Conceptualization:** BaiSong Yu, Chunxia Dong, Zhijuan Hu, Bing Liu.

**Data curation:** BaiSong Yu, Chunxia Dong, Zhijuan Hu.

**Formal analysis:** BaiSong Yu, Chunxia Dong, Zhijuan Hu.

**Investigation:** BaiSong Yu, Chunxia Dong.

**Methodology:** BaiSong Yu, Chunxia Dong, Bing Liu.

Project administration: BaiSong Yu.

**Resources:** BaiSong Yu.

**Software:** BaiSong Yu.

**Supervision:** BaiSong Yu, Chunxia Dong, Zhijuan Hu, Bing Liu.

**Validation:** Zhijuan Hu, Bing Liu.

**Visualization:** Zhijuan Hu, Bing Liu.

**Writing – original draft:** BaiSong Yu, Chunxia Dong, Zhijuan Hu, Bing Liu.

**Writing – review & editing:** BaiSong Yu, Chunxia Dong, Zhijuan Hu, Bing Liu.

## References

[1] Ogurtsova K da Rocha Fernandes JD Huang Y. IDF Diabetes Atlas: Global estimates for the prevalence of diabetes for 2015 and 2040. Diabetes Res Clin Pract 2017;128:40–50. 28437734 10.1016/j.diabres.2017.03.024

[2] de Boer IH Rue TC Hall YN. Temporal trends in the prevalence of diabetic kidney disease in the United States. JAMA 2011;305:2532–9. 21693741 10.1001/jama.2011.861 PMC3731378

[3] Shahbazian H Rezaii I. Diabetic kidney disease; review of the current knowledge. J Renal Inj Prev 2013;2:73–80. 25340133 10.12861/jrip.2013.24 PMC4206005

[4] Tomino Y Gohda T. The Prevalence and Management of Diabetic Nephropathy in Asia. Kidney Dis (Basel) 2015;1:52–60. 27536665 10.1159/000381757 PMC4934822

[5] Bilous R Chaturvedi N Sjolie AK. Effect of candesartan on microalbuminuria and albumin excretion rate in diabetes: three randomized trials. Ann Intern Med 2009;151:11–20. 19451554 10.7326/0003-4819-151-1-200907070-00120

[6] Baker WL Smyth LR Riche DM. Effects of sodium-glucose co-transporter 2 inhibitors on blood pressure: a systematic review and meta-analysis. J Am Soc Hypertens 2014;8:262–75. 24602971 10.1016/j.jash.2014.01.007

[7] Matthaei S Bowering K Rohwedder K. Dapagliflozin improves glycemic control and reduces body weight as add-on therapy to metformin plus sulfonylurea: a 24-week randomized, double-blind clinical trial. Diabetes Care 2015;38:365–72. 25592197 10.2337/dc14-0666

[8] Neal B Perkovic V Matthews DR. Canagliflozin and cardiovascular and renal events in type 2 diabetes. N Engl J Med 2017;377:644–57. 29166232 10.1056/NEJMc1712572

[9] Wiviott SD Raz I Bonaca MP. Dapagliflozin and cardiovascular outcomes in type 2 diabetes. N Engl J Med 2019;380:347–57. 30415602 10.1056/NEJMoa1812389

[10] Thomson SC Rieg T Miracle C. Acute and chronic effects of SGLT2 blockade on glomerular and tubular function in the early diabetic rat. Am J physiol Regul integr comp physiol 2012;302:R75–83. 21940401 10.1152/ajpregu.00357.2011 PMC3349378

[11] M C.T C D.Z.I. The actions of SGLT2 inhibitors on metabolism, renal function and blood pressure. Diabetologia 2018;61:2098–107. DOI: 10.1007/s00125-018-4669-0. 30132034 10.1007/s00125-018-4669-0

[12] Tonneijck L Muskiet MH Smits MM. Glomerular hyperfiltration in diabetes: mechanisms, clinical significance, and treatment. J Am Soc Nephrol 2017;28:1023–39. 28143897 10.1681/ASN.2016060666 PMC5373460

[13] Lytvyn Y Bjornstad P Udell JA. Sodium glucose cotransporter-2 inhibition in heart failure: potential mechanisms, clinical applications, and summary of clinical trials. Circulation 2017;136:1643–58. 29061576 10.1161/CIRCULATIONAHA.117.030012 PMC5846470

[14] Bessho R Takiyama Y Takiyama T. Hypoxia-inducible factor-1alpha is the therapeutic target of the SGLT2 inhibitor for diabetic nephropathy. Sci Rep 2019;9:14754 DOI: 10.1038/s41598-019-51343-1. 31611596 10.1038/s41598-019-51343-1 PMC6791873

[15] Inoue M Matsunaga Y Nakatsu Y. Possible involvement of normalized Pin1 expression level and AMPK activation in the molecular mechanisms underlying renal protective effects of SGLT2 inhibitors in mice. Diabetol metab syndr 2019;11:57 DOI: 10.1186/s13098-019-0454-6. 31367234 10.1186/s13098-019-0454-6 PMC6647324

[16] Perkovic V Jardine MJ Neal B. Canagliflozin and renal outcomes in type 2 diabetes and nephropathy. N Engl J Med 2019;380:2295–306. 30990260 10.1056/NEJMoa1811744

[17] Wanner C Inzucchi SE Zinman B. Empagliflozin and progression of kidney disease in type 2 diabetes. N Engl J Med 2016;375:323–34. 27299675 10.1056/NEJMoa1515920

[18] Moher D Shamseer L Clarke M. Preferred reporting items for systematic review and meta-analysis protocols (PRISMA-P) 2015: elaboration and explanation. Syst Rev 2015;350:g7647 DOI: 10.1186/2046-4053-4-1. 10.1136/bmj.g7647 25555855

[19] Takashima H Yoshida Y Nagura C. Renoprotective effects of canagliflozin, a sodium glucose cotransporter 2 inhibitor, in type 2 diabetes patients with chronic kidney disease: a randomized open-label prospective trial. Diab Vasc Dis Res 2018;15:469–72. 29923427 10.1177/1479164118782872

[20] Yale JF Bakris G Cariou B. Efficacy and safety of canagliflozin over 52 weeks in patients with type 2 diabetes mellitus and chronic kidney disease. Diabetes Obes Metab 2014;16:1016–27. 24965700 10.1111/dom.12348

[21] Bode B Stenlöf K Harris S. Long-term efficacy and safety of canagliflozin over 104 weeks in patients aged 55–80 years with type 2 diabetes. Diabetes Obes Metab 2015;17:294–303. 25495720 10.1111/dom.12428

[22] Kohan DE Fioretto P Tang W. Long-term study of patients with type 2 diabetes and moderate renal impairment shows that dapagliflozin reduces weight and blood pressure but does not improve glycemic control. Kidney Int 2014;85:962–71. 24067431 10.1038/ki.2013.356 PMC3973038

[23] Wilding JP Woo V Soler NG. Long-term efficacy of dapagliflozin in patients with type 2 diabetes mellitus receiving high doses of insulin: a randomized trial. Ann Intern Med 2012;156:405–15. 22431673 10.7326/0003-4819-156-6-201203200-00003

[24] Barnett AH Mithal A Manassie J. Efficacy and safety of empagliflozin added to existing antidiabetes treatment in patients with type 2 diabetes and chronic kidney disease: a randomised, double-blind, placebo-controlled trial. Lancet Diabetes Endocrinol 2014;2:369–84. 24795251 10.1016/S2213-8587(13)70208-0

[25] Allegretti AS Zhang W Zhou W. Safety and effectiveness of bexagliflozin in patients with type 2 diabetes mellitus and stage 3a/3b CKD. Am J Kidney Dis 2019;74:328–37. 31101403 10.1053/j.ajkd.2019.03.417 PMC10077840

[26] Xu L Li Y Lang J. Effects of sodium-glucose co-transporter 2 (SGLT2) inhibition on renal function and albuminuria in patients with type 2 diabetes: a systematic review and meta-analysis. Peer J 2017;5:e3405 DOI: 10.7717/peerj.3405. 28663934 10.7717/peerj.3405 PMC5490461

[27] Cahn A Melzer-Cohen C Pollack R. Acute renal outcomes with sodium-glucose co-transporter-2 inhibitors: real-world data analysis. Diabetes Obes Metab 2019;21:340–8. 30207040 10.1111/dom.13532

